# PD-1 Controls Tonic Signaling and Lymphopenia-Induced Proliferation of T Lymphocytes

**DOI:** 10.3389/fimmu.2017.01289

**Published:** 2017-10-12

**Authors:** Kristofor K. Ellestad, Jiaxin Lin, Louis Boon, Colin C. Anderson

**Affiliations:** ^1^Department of Medical Microbiology and Immunology, University of Alberta, Edmonton, AB, Canada; ^2^Alberta Diabetes Institute, University of Alberta, Edmonton, AB, Canada; ^3^Department of Surgery, University of Alberta, Edmonton, AB, Canada; ^4^Bioceros B.V., Utrecht, Netherlands; ^5^Alberta Transplant Institute, University of Alberta, Edmonton, AB, Canada

**Keywords:** tolerance, autoimmunity, co-stimulation, co-inhibition, lymphopenia, PD-1, mouse models, IL-7

## Abstract

Recovery of the T lymphocyte compartment within a lymphopenic host by lymphopenia-induced proliferation (LIP) is regulated by inter- and intraclonal competition for limited resources, including homeostatic cytokines and peptide:MHC (pMHC) complexes with which the TCR can interact at least weakly to yield a tonic signal. Importantly, the process of LIP can synergize with other factors that promote T cell activation to drive inflammatory disease. While reconstitution of the lymphoid compartment of immune deficient Rag^−/−^ mice by transfer of wild-type hematopoietic stem cells (HSC) does not generally result in an overt disease phenotype, transfer of HSC deficient in expression of the co-inhibitory molecule PD-1 results in severe systemic autoimmunity driven by newly generated T cells that emerge from the thymus into the periphery and undergo LIP. Importantly, autoimmunity does not appear to depend on a response to exogenous (i.e., gut flora-derived) antigens. PD-1 is well known to be upregulated during T cell activation in response to cognate antigens, but it is unclear whether PD-1 has a role in controlling LIP of T cells in the absence of cognate antigen, i.e., in response to tonic pMHC. We examined whether PD-1 controls LIP of newly generated T cells by controlling the response to tonic pMHC or the homeostatic cytokine IL-7. We found that PD-1-deficient T cells have a proliferative advantage over WT T cells during LIP and this effect is MHC-II dependent and independent of IL-7Rα signaling. Furthermore, our data suggest that signals through IL-7Rα can be dispensable for LIP and may instead be of increased importance for T cell survival in conditions of high competition for limited pMHC (e.g., post-LIP, in a lymphoreplete host). We hypothesize that autoimmunity post-PD-1^−/−^ HSC transplant is the result of an overzealous T cell response to normally tonic self-pMHC precipitated by the synergy of LIP and PD-1 deficiency. Furthermore, potentiation of TCR signals in response to normally tonic self-pMHC may contribute to the success of PD-1 blockade in cancer immunotherapy.

## Introduction

The number of cells in and diversity of the peripheral T lymphocyte pool is controlled by intra- and interclonal competition for resources, which together define T cell “space” (1). Such resources include homeostatic cytokines like IL-7 and IL-15, but also peptide:MHC (pMHC) complexes; often self-pMHC, with which the TCR can interact and receive at least a weak “tonic” signal to promote T cell survival. In lymphopenic hosts, recovery of the T cell compartment occurs *via* a process known as lymphopenia-induced proliferation (LIP), where resources are in excess and T cells expand to fill the available niche. While the term lymphopenia lacks a precise quantitative character, one can consider the extent to which LIP can occur, or “LIP potential” of a host, as a ratio of the available resources to the number of competitors for those resources (2). Thus, provision of competition for a particular pMHC can reduce LIP potential for other T cells that recognize the same pMHC (3–6). Similarly, Treg, which may be viewed as “super”-competitors for pMHC, can inhibit LIP (5, 7–14). Positive or negative regulation of the strength of the TCR signal a lymphocyte receives in response to a given pMHC, for example by blocking/reducing co-inhibition (15–19) or co-stimulation (9), respectively, can modulate LIP potential. Finally, a neonatal host might be viewed to have a low LIP potential due to small anatomic size (low absolute quantities of resources) as well as the recently described presence of an innate lymphoid cell population that may act as a sink for IL-7 (20).

Importantly, LIP is strongly associated with acquisition of an effector-memory phenotype in T cells, including the ability to rapidly mediate effector function (21), and in the context of concurrent infections or deficiencies of co-inhibitory pathways, LIP can result in overzealous T cell responses and autoimmune disease (2, 7, 14, 17, 19, 22–30). This association between LIP and inappropriate inflammation may be rationalized by considering that the conditions present in lymphopenia—including reduced competition for access to a given T cell’s cognate or tonic pMHC, along with high concentrations of IL-7 and IL-15 [which have been suggested to potentiate TCR signals (31, 32)]—may conspire to increase the frequency with which T cells can productively interact with pMHC and generate an abnormally strong TCR signal when they do so. Systemic activation of sufficient numbers of T cells may result in immune pathology.

Reconstitution of the lymphoid compartment of an otherwise normal C57BL/6 (B6) Rag^−/−^ mouse with B6 hematopoietic stem cells (HSC) does not typically result in the development of apparent autoimmune disease (19, 33). In stark contrast, we recently demonstrated that reconstitution of adult Rag^−/−^ mice with HSC deficient in the co-inhibitory molecule PD-1 results in a rapid, severe, and frequently lethal systemic autoimmune disease soon after the first newly generated T cells emerge from the thymus (19). Severe and rapid autoimmunity can also result from transfer of PD-1^−/−^ thymocytes to Rag^−/−^ recipients, but not established peripheral cells from adult PD-1^−/−^ mice [note, on the B6 background, PD-1^−/−^ mice develop only a mild lupus-like autoimmune disease upon aging (34)]. The newly generated T cell population may have a higher average affinity for self-pMHC compared to established cells that developed in a lymphoreplete adult environment or an environment with low LIP potential (e.g., during early seeding of the lymphoid compartment in the neonate) and thus underwent more normal peripheral tolerance mechanisms (deletion, anergy, conversion to pTreg). One hypothesis to explain disease in our model is that in the “three-strikes” scenario of high LIP potential, PD-1 deficiency, and a periphery seeded with newly generated T cells, a stochastically greater number of the T cells on the higher end of the self-pMHC affinity spectrum receive a strong enough signal to acquire effector function giving rise to the observed systemic cytokinemia (19). On the other hand, when PD-1 is sufficient, its restraint on the magnitude of the TCR signal (35–37) limits the frequency of such spurious activation preventing a pathological systemic effect. Although multiple co-inhibitory molecules other than PD-1 have been shown to control LIP (15–18) and our data suggest that PD-1 does as well (19, 33), whether they do so *in vivo* by controlling TCR signals mediated by tonic self-pMHC is unknown. Indeed, PD-1 is known to be upregulated on *bona fide* activated T cells and it is unclear whether the generally weak, tonic signals provided by interaction with self-pMHC can drive its expression. Co-inhibitor deficient cells might instead come to predominate in LIP due to control of responses to homeostatic cytokines or completely independent of external signals. Furthermore, because of the polyclonal repertoire generated post-HSC transplant in our disease model, we cannot rule out that the response might be directed to foreign pMHC [although depletion of gut microbiota in PD-1^−/−^ HSC recipients does not protect from disease (33)] or pMHC derived from tissue-restricted antigens against which developing PD-1^−/−^ T cells failed to be appropriately negatively selected in the thymus. Herein, we examined whether PD-1 is able to control the response to tonic pMHC using transfer of monoclonal PD-1^+/+^ or PD-1^−/−^ male antigen (HY)-specific Marilyn CD4^+^ TCR transgenic T cells (38) to MHC-II sufficient and deficient [MHC Class II transactivator (CiiTA) knockout (39)] lymphopenic hosts lacking the male antigen HY. We also examined whether PD-1 modulates signaling through IL-7Rα during LIP. We found that PD-1 controls pMHC-dependent tonic signals to T cells, independent of IL-7 signaling. Furthermore, our data suggest that IL-7 is particularly important for controlling T cell homeostasis during situations of high competition for limited pMHC but is not essential for LIP when available pMHC is abundant.

## Materials and Methods

### Mice

B6.129S7-*Rag1*^tm1Mom^/J (Rag1^−/−^, Rag^−/−^), B6.Cg-*Foxp3*^tm2(EGFP)Tch^/J (FoxP3^EGFP^), B6.SJL-Ptprca Pepcb/BoyJ (CD45.1), and B6.129S2-*Ciita*^tm1Ccum^/J (CiiTA^−/−^) mice were originally purchased from The Jackson Laboratory (Bar Harbor, ME, USA) and then bred at the University of Alberta. C57BL/6-*Pdcd1*^−/−^ (backcrossed 11 generations to C57BL/6) were originally generated by Prof. T. Honjo and colleagues (34). FoxP3^EGFP^ × *Pdcd1*^−/−^ mice were generated by crossing the above FoxP3^EGFP^ and B6-*Pdcd1*^−/−^ mice and are referred to and used in the present manuscript simply as PD-1^−/−^. Rag^−/−^ × CiiTA^−/−^ mice were generated by crossing the above Rag^−/−^ and CiiTA^−/−^ mice. Marilyn Rag2^−/−^ CD4^+^ anti-IA^b^-HY TCR transgenic mice (called Marilyn herein) were originally generated by Lantz and colleagues (38) and were originally obtained from the NIAID exchange program. Marilyn-CD45.1, referred to herein as Marilyn-WT, were generated by crossing the above Marilyn mice with CD45.1 × Rag2^−/−^ mice (Taconic). Marilyn-PD-1^−/−^ mice were generated by crossing the above Marilyn mice with PD-1^−/−^ mice and were maintained as Rag2^−/−^. All Marilyn mice used as donors were female. Animals were cared for in accordance with the guidelines of the Canadian Council on Animal Care and housed under clean conventional housing conditions at the University of Alberta Health Sciences and Laboratory Animal Services facilities (HSLAS).

### Cell Preparations and Adoptive Transfer Experiments

For experiments involving transfer of thymocytes, recipient NK cells were depleted [to avoid potential NK-mediated killing of the input cells (40–42)] by treatment on days −4, −1, and +2 with 0.3 mg per mouse of anti-NK1.1 (PK136) injected intraperitoneally. In order to study the role of PD-1 in controlling LIP of newly generated T cells, which would not have been exposed to the periphery where they may have undergone peripheral tuning mechanisms, we chose to transfer minimally manipulated whole unfractionated thymocytes and monitor their proliferation *in vivo*. In mixed thymocyte transfer experiments, WT (CD45.1^+^) and PD-1^−/−^ (CD45.1−) Marilyn unfractionated thymocytes were counted and mixed in equal proportions prior to labeling and transfer. Labeling of mixed thymocyte populations with Celltrace violet (CTV, ThermoFisher) was performed using 5 µM CTV according to the manufacturer’s protocols except with a final cell concentration in the labeling reaction of up to ~40 × 10^6^ cells per mL. For subsequent *in vivo* use cells were washed once with PBS and resuspended in PBS on ice for immediate intravenous tail vein injection into recipient mice. Mixed cells for infusion into mice were analyzed by flow cytometry to determine the starting (day 0) ratio of PD-1^−/−^: WT T cells calculated as %PD-1^−/−^ T cells divided by % WT T cells. Initial ratios varied from 0.57 to 1.09. To facilitate combining data from multiple experiments, initial ratios were normalized to 1 by multiplying by a scaling factor which was applied to all subsequent PD-1^−/−^:WT T cell ratio measurements within a given experiment. Anti-IL-7Rα treated mixed thymocyte recipients received twice weekly intraperitoneal injections of antibody (as described below) for the full course of the experiment.

For *in vitro* restimulation assays, cells were resuspended in E-DMEM (high glucose DMEM + 2 mM l-glutamine, 1 mM sodium pyruvate, 0.1 mM nonessential amino acids, 100 U/mL penicillin, 0.1 mg/mL streptomycin, 50 µM 2-mercaptoethanol, 10% FBS) at 2 × 10^6^ cells/mL and seeded 200 µL per well into a 96 well, round bottom plate. A final concentration of 16 nM of phorbol 12-myristate 13-acetate (PMA, Sigma-Aldrich) and 1.4 µM ionomycin (Sigma-Aldrich) were added for 2 h, at which time brefeldin A (eBioscience, 3 µg/mL) and monensin (eBioscience, 2 µM final) were added for a further 2 h prior to surface staining, fixation, intracellular staining, and analysis.

### Definition of Disease and Data Analysis

Macroscopic signs of disease in thymocyte recipients included cachexia/weight loss (>15%), kyphosis (hunched appearance), ruffled fur, dermatitis, ocular lesions, and diarrhea. Recipient mice were no longer considered disease free when two or more of the above symptoms were evident, or if mice lost ≥20% body weight. Kaplan–Meier survival curve analysis of disease onset/incidence was performed using Graphpad Prism v5.0 software. Probability values reported for survival curve comparisons were calculated using the Mantel-Cox method. For thymocyte experiments, calculation of weight loss for disease determination was performed relative to weights at day 0 or day 1 relative to cell transfer. Unless otherwise indicated, animals were used between 6 and 20 weeks of age.

### Antibodies and Flow Cytometry

For flow cytometric staining, fluorophore-labeled antibodies against the following markers were obtained from eBioscience (San Diego, CA, USA) unless otherwise indicated: CD4 (RM4-5), TCRβ (H57-597), CD8 (53-6.7), PD-1 (J43), CD5 (53-7.3), IL-7Rα/CD127 (A7R34), IFN-γ (XMG1.2), Bcl2 (10C4). Antibodies were used at manufacturer’s recommended concentrations. Flow cytometric staining always used an Fc block cocktail to block nonspecific staining. Fc block cocktail consisted of 3 mL each of normal mouse, rat, and hamster serum, with addition of 0.3 mg of anti-CD16/32 antibody (clone 2.4g2, BioXCell). For intracellular staining, cells were fixed and permeabilized using the eBioscience FoxP3 Fixation/permeabilization buffer kit according to supplied protocols. Standard flow cytometric analysis was performed using a BD LSR II instrument. Flow cytometric data analysis was performed using FlowJo (Treestar software, Portland, OR, USA).

Anti-IL-7Rα treatment *in vivo* was carried out by biweekly intraperitoneal injection of 0.5 mg of anti-IL-7Rα clone A7R34 (BioXcell for polyclonal PD-1^−/−^ thymocyte disease model experiments; or generated by us for mixed Marilyn experiments) or Rat IgG2a isotype control (2A3, BioXcell or generated by us) beginning on the day of cell transfer.

### Statistical Analyses

For statistical analyses, we used Graphpad Prism software. Unless otherwise noted Student’s *t*-test was used for comparisons. In the case of unequal variances, *t*-test was performed using Welch’s correction. For comparisons of multiple groups, one-way ANOVA with Tukey’s multiple comparison test was used.

## Results

### PD-1 Controls LIP in Response to Tonic Self-pMHC-II Independent of IL-7 Signals

**Figure 1:**
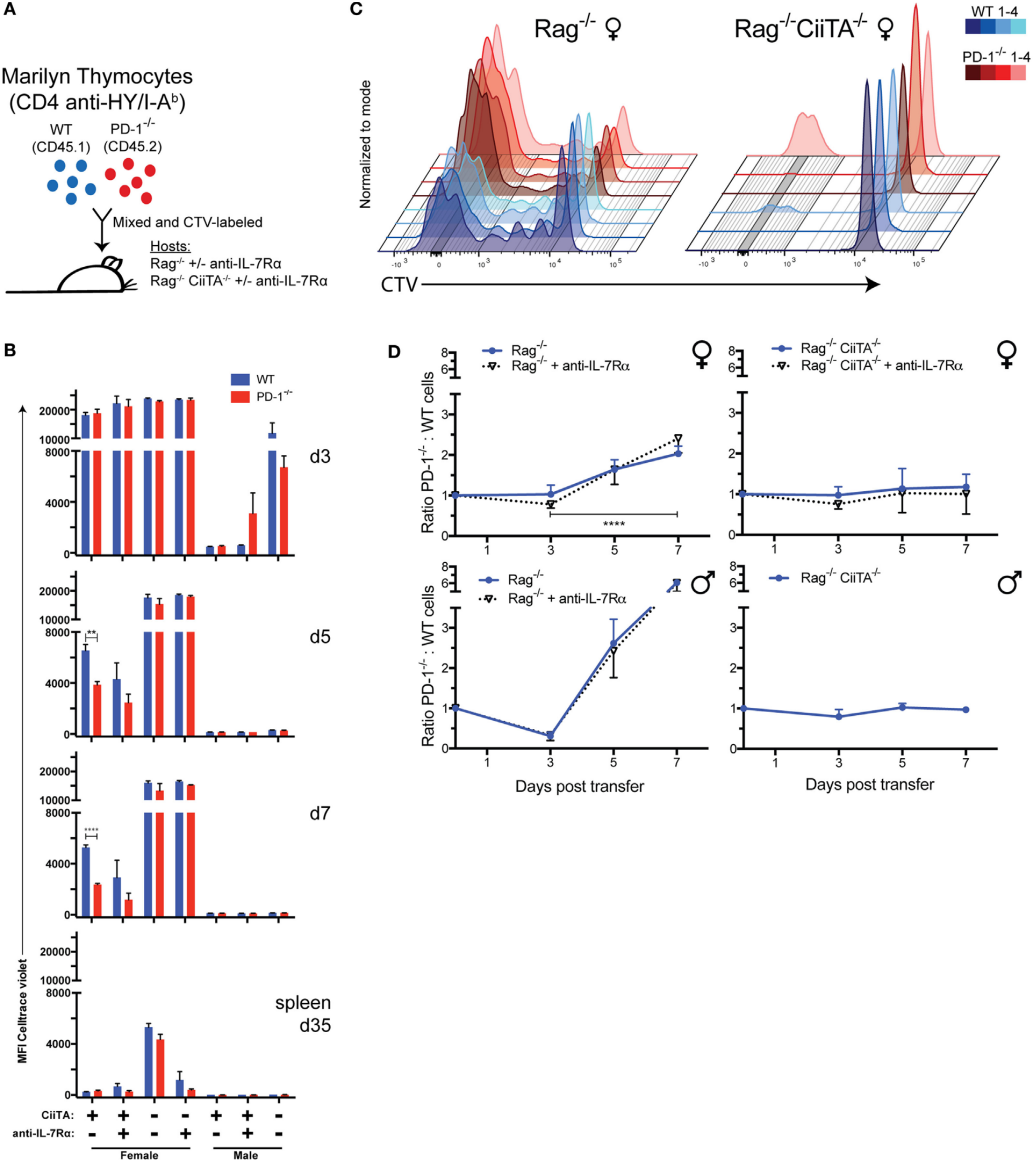
Monoclonal CD4^+^ HY-specific PD-1^−/−^ T cells out-proliferate and outnumber WT cells during LIP in response to tonic or cognate pMHC-II but independent of IL-7 signals. **(A)** 40 × 10^6^ Celltrace violet (CTV)-labeled mixed Marilyn-WT (CD45.1^+^) and PD-1^−/−^ (CD45.2^+^) thymocytes were transferred i.v. to Rag^−/−^ or CiiTA^−/−^ × Rag^−/−^ male or female hosts with or without anti-IL-7Rα treatment. **(B)** Mean fluorescence intensity (MFI) of CTV in TCRβ^+^ CD4^+^ WT or PD-1^−/−^ cells from blood collected at 3, 5, and 7 days and spleen at 35 days post-transfer (p.t.) ± SEM, *n* = 3–4 per group. **p < 0.01, ****p < 0.0001, Student’s *t*-test. **(C)** Overlaid flow cytometry plots of CTV fluorescence at 7 days p.t. of mixed WT and PD-1^−/−^ Marilyn thymocytes to Rag^−/−^ and CiiTA^−/−^ × Rag^−/−^ female hosts. Four recipients from one experiment are depicted – with color darkness indicating recipient identity and blue and red hues representing WT and PD-1^−/−^ cells, respectively, in a given recipient. **(D)** Ratio of PD-1^−/−^ to WT CD4^+^ T cells in blood among indicated recipients at day 3–7 p.t. ± SEM. Data presented are combined from multiple independent experiments as follows: female Rag^−/−^ (four independent experiments, *n* = 12, 16, 13 per group at days 3, 5, 7 respectively), female Rag^−/−^ + anti-IL-7Rα (two independent experiments, *n* = 4, 8, 8 at days 3, 5, 7), female CiiTA^−/−^ × Rag^−/−^ (four independent experiments, *n* = 9, 11, 10 at days 3, 5, 7), female CiiTA^−/−^ × Rag^−/−^ + anti-IL-7Rα (two independent experiments, *n* = 3, 5, 5 at days 3, 5, 7), male Rag^−/−^ (two independent experiments, *n* = 8), male Rag^−/−^ + anti-IL-7Rα (two independent experiments, *n* = 7), male CiiTA^−/−^ × Rag^−/−^ (two independent experiments, *n* = 8, 7, 8 at days 3, 5, 7). ****p < 0.0001, Student’s *t*-test, Rag^−/−^ day 3 versus day 7. The starting ratio was normalized and set to a value of one with subsequent ratio measurements scaled accordingly.

We have previously demonstrated that transfer of a mixture of Treg-depleted polyclonal WT and PD-1^−/−^ CD4^+^ T cells purified from the thymocyte or splenocyte population (including CD62L^hi^ selected cells) into lymphopenic Rag^−/−^ hosts resulted in predominance of the PD-1^−/−^ population in the periphery during LIP (33). In the same study, we also demonstrated that the proportion of WT and PD-1^−/−^ cells could be equalized by treatment of hosts with anti-PD-L1, releasing the WT population from cell-intrinsic PD-1-dependent inhibition. These data indicated that the effect on population size was mediated through the PD-L1:PD-1 interaction. In order to examine whether PD-1 controls LIP in response to tonic self-pMHC signals in the absence of potential responses to conventional agonist cognate antigens by PD-1^−/−^ cells within a polyclonal repertoire, we employed Marilyn male antigen-specific CD4^+^ TCR transgenic CD45.1 (CD45.1^+^, “Marilyn-WT”) mice as well as Marilyn PD-1^−/−^ mice (CD45.2^+^, Marilyn-PD-1^−/−^) (43). We also generated Rag^−/−^ and MHC Class II transactivator deficient (CiiTA^−/−^) mice, which are largely MHC-II deficient (39) (CiiTA^−/−^ × Rag^−/−^ mice). We transferred a mixture (40 × 10^6^ cells) of Marilyn-WT and Marilyn-PD-1^−/−^ thymocytes labeled with CTV proliferation dye into NK-depleted Rag^−/−^ females (tonic signals only) or males (systemic cognate HY antigen present) and CiiTA^−/−^ × Rag^−/−^ females or males, with or without biweekly anti-IL-7Rα treatment and monitored their relative proliferation and abundance over time (Figure 1A). NK depletion was carried out to maximize input cell survival as even syngeneic cells can be targets of NK cell killing (40–42).

Early after transfer (day 3), only barely detectable levels of proliferation had occurred within female Rag^−/−^ recipients with none in the female Rag^−/−^ anti-IL-7Rα-treated group (Figure 1B; Figure S1 in Supplementary Material), and no difference between the WT and PD-1^−/−^ cells was apparent nor was there any change in the ratio of PD-1^−/−^ to WT T cells in these recipients relative to the initial seeding ratio, regardless of anti-IL-7Rα treatment (Figure 1D). In contrast, in the presence of systemic cognate antigen all of the cells within the Rag^−/−^ males, regardless of ant-IL-7Rα treatment, had proliferated extensively and had already almost completely diluted out their CTV dye (Figure 1B; Figure S1 in Supplementary Material). Proliferation had also clearly occurred albeit to a reduced extent within CiiTA^−/−^ × Rag^−/−^ male recipients, which may be due to small numbers of MHC-II sufficient thymic DC that were transferred along with the thymocytes or the known residual MHC-II expression in these knockouts (39, 44).

**Figure 2:**
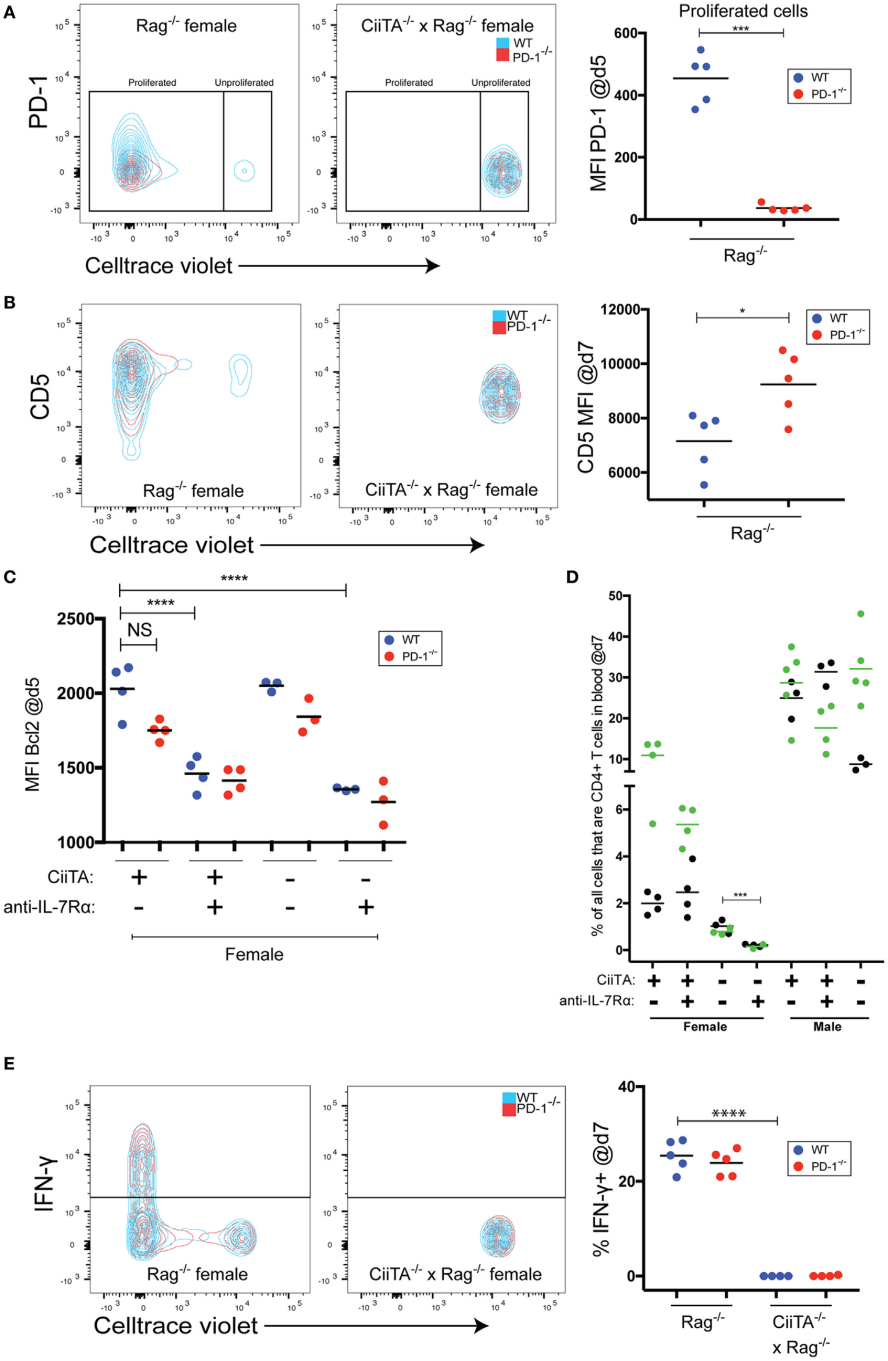
Tonic peptide:MHC signals control expression of co-inhibitory receptors, drive IFNγ expression, and limit the need for IL-7 signals during LIP. **(A)** (Left and center panels) Representative flow cytometry overlaid contour plots showing PD-1 expression versus. Celltrace violet (CTV) staining within the indicated recipients of 40 × 10^6^ mixed Marilyn-WT and PD-1^−/−^ thymocytes at day 5 p.t. in blood. (Right panel) Mean fluorescence intensity (MFI) of PD-1 staining within the proliferated WT or PD-1^−/−^ cell populations from individual recipients. No significant difference in PD-1 expression was noted in unproliferated WT or PD-1^−/−^ cells in CiiTA^−/−^ × Rag^−/−^ hosts. ***p < 0.001, Student’s *t*-test. **(B)** (Left and center panels) Representative overlaid contour plots of CD5 expression versus CTV staining in the indicated recipients of mixed Marilyn-WT and PD-1^−/−^ cells at day 7 p.t. in blood. (Right panel) MFI of CD5 staining within WT and PD-1^−/−^ populations from individual recipients. *p < 0.05, Student’s *t*-test. **(C)** Mean fluorescence intensity of Bcl2 staining in WT and PD-1^−/−^ CD4^+^ T cell populations in the blood at day 5 p.t. within the indicated recipients. ****p < 0.0001, One-way ANOVA with Tukey’s Multiple comparison test. Data in **(C)** depicts individual biological replicates from one experiment. **(D)** Comparison of the % of all cells that were CD4^+^ T cells in blood of indicated recipient groups at day 7 p.t. ***p < 0.001, Student’s *t*-test. Two independent experiments are depicted with black (experiment 1) and green (experiment 2) symbols and means. **(E)** (Left and center panels) Representative overlaid contour plots of IFN-γ expression versus. CTV staining in indicated recipients at day 7 p.t. in *in vitro* restimulated splenocytes. (Right panel) % IFN-γ^+^ cells within WT and PD-1^−/−^ CD4^+^ T cell populations from individual recipients. ****p < 0.0001, Student’s *t*-test. For panels **(A,B,E)**, data presented are from individual recipients from one experiment that is representative of two independent experiments.

By day 5, PD-1^−/−^ T cells had undergone significantly more proliferation than WT cells in Rag^−/−^ female recipients (Figure 1B; Figure S1 in Supplementary Material) and the ratio of PD-1^−/−^:WT cells started to increase (Figure 1D). Furthermore, in these recipients, we detected an MHC-II-dependent upregulation of PD-1 expression within the WT cell population at this time point among the extensively proliferated cells (Figure 2A), indicating that tonic pMHC signals are sufficient to upregulate PD-1. We also explored expression of another co-inhibitory receptor, CD5, a commonly used marker of TCR affinity and that has co-inhibitory function (45–47), and found it was modulated during LIP in response to tonic pMHC signals. Upon extensive proliferation, CD5 became up or downregulated on a substantial proportion of WT cells, while PD-1^−/−^ cells had less downregulation of CD5 (Figure 2B), suggesting CD5 expression may help compensate for the lack of PD-1.

By 7 days post-cell transfer, the majority of cells within the Rag^−/−^ female recipients had proliferated extensively and diluted their CTV labeling beyond detection limits, although significantly more proliferation had occurred in the PD-1^−/−^ cell population in these recipients as judged by comparison of CTV MFI (Figures 1B,C; Figure S1 in Supplementary Material). Furthermore, the ratio of PD-1^−/−^:WT cells in these animals increased approximately twofold relative to the input cell ratio which was significantly different from the female CiiTA^−/−^ × Rag^−/−^ recipients (2.03 ± 0.05 versus 1.18 ± 0.10, *p* < 0.0001, Figure 1D) in which the PD-1:WT ratio did not change and also in which the majority of the T cell population still had not proliferated (Figures 1B,C; Figure S1 in Supplementary Material). Importantly, this alteration in ratio was not affected by anti-IL-7Rα treatment of the hosts. During LIP 7 days post-mixed thymocyte transfer, the percentage of all cells that were TCRβ^+^CD4^+^ in blood of Rag^−/−^ female recipients was not significantly affected by anti-IL-7Rα treatment; although, we did note a trend to reduced CD4 T cells with anti-IL-7Rα in the one experiment in which CD4 numbers were already quite high by day 7 (Figure 2D, green symbols). In contrast, in MHC-II-deficient female CiiTA × Rag^−/−^ recipients, blockade of anti-IL-7Rα significantly reduced the frequency of CD4^+^ T cells in the blood. Examination of the CTV dilution histograms suggested that anti-IL-7Rα treatment in the MHC-II sufficient female Rag^−/−^ recipients appeared to primarily decrease the size of the unproliferated cell population (Figure S1 in Supplementary Material). In the presence of HY antigen in male Rag^−/−^ recipients, by day 7 the ratio of PD-1^−/−^:WT cells had increased more than sixfold relative to the input cell proportions, and this was also not affected by anti-IL-7Rα treatment (Figure 1D). Interestingly, the ratio of PD-1^−/−^:WT cells did not change by day 7 in CiiTA^−/−^ × Rag^−/−^ male recipients despite robust expansion of input populations in these recipients; expansion that was potentially caused by residual MHC class II known to exist in the CiiTA knockout (39, 44). Male Rag^−/−^ recipients had a significantly higher peripheral blood T cell abundance compared to females which was not significantly decreased by anti-IL-7Rα treatment (Figure 2D).

The increased proportion of PD-1^−/−^ cells during tonic pMHC-stimulated LIP could have been due to increased proliferation or increased survival of proliferating cells. However, PD-1^−/−^ cells did not have an increase of the pro-survival molecule Bcl2 (Figure 2C). In addition, Bcl2 expression, known to be stimulated by IL-7 signals (48), generally appeared to be depressed in response to anti-IL-7Rα treatment (Figure 2C) despite anti-IL-7Rα’s lack of effect on both LIP (Figure 1B) and CD4 T cell numbers in MHC-II positive recipients (Figure 2D). However, IL-7Rα blockade together with a lack of MHC-II led to the strongest decrease in Bcl2 expression in both WT and PD-1^−/−^ cells (Figure 2C), and this was associated with reduced CD4 T cell numbers (Figure 2D). Thus, IL-7 and Bcl2 appear important for CD4 T cells in the absence of tonic pMHC signals but not during tonic pMHC-stimulated LIP. Together, these data indicate that PD-1 controls signaling in response to tonic as well as cognate pMHC-II in a cell-intrinsic manner, which is independent of effects on IL-7 signaling.

Thus far, we have shown that PD-1 controls LIP to tonic pMHC signals. Whether tonic pMHC signals driven effector function (e.g., cytokine production) in addition to LIP is controlled by PD-1 is not known. We found no difference in the proportion of WT versus PD-1^−/−^ T cells producing IFN-γ in response to *in vitro* restimulation at the d7 time point in the MHC-II sufficient female hosts, and in the absence of MHC-II IFN-γ was essentially undetectable (Figure 2E). Thus, while tonic pMHC signals are able to stimulate IFN-γ production, PD-1 appears important for controlling proliferation but not this effector activity triggered by tonic pMHC signals in LIP. However, it remains possible that PD-1 controls other effector functions of CD4 T cells during LIP.

### IL-7 Receptor Blockade Decreases T Cell Compartment Size in Conditions of Limiting pMHC

**Figure 3:**
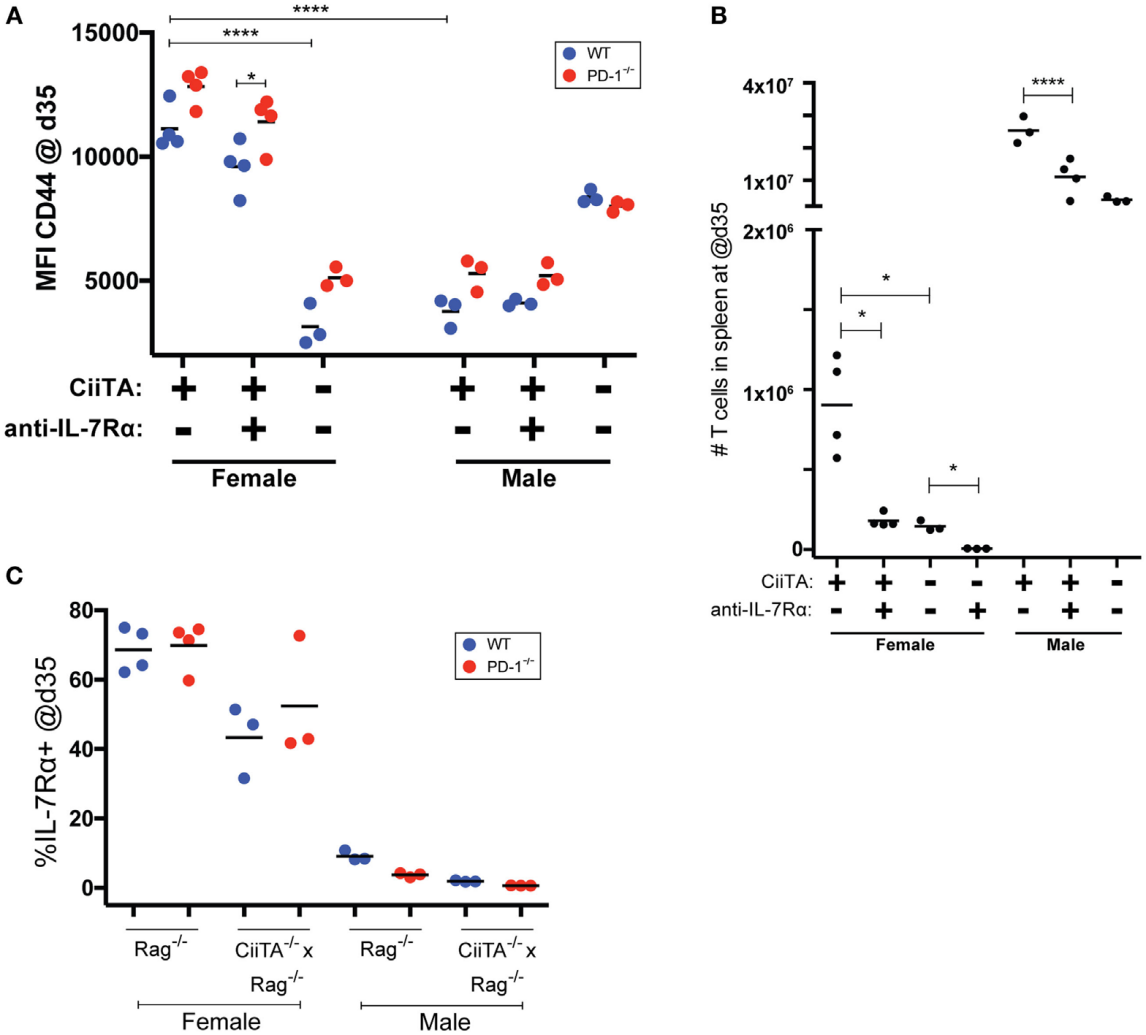
IL-7Rα blockade decreases T cell compartment size post-LIP without preventing tonic peptide:MHC induced upregulation of CD44. **(A)** Mean fluorescence intensity (MFI) of CD44 expression in TCRβ^+^CD4^+^ WT or PD-1^−/−^ cells in splenocytes from individual recipients collected at 35 days p.t. (post-LIP). *p < 0.05, ****p < 0.0001, One-way ANOVA with Tukey’s multiple comparison test. **(B)** Absolute numbers of CD4^+^ T cells in spleen of recipient mice at day 35 p.t. *p < 0.05, Student’s *t*-test, ****p < 0.0001 One-way ANOVA with Tukey’s multiple comparison test. **(C)** Percent IL-7Rα^+^ cells within the WT and PD-1^−/−^ CD4^+^ T cell populations in the indicated recipients’ splenocytes at day 35 p.t. The data depict individual biological replicates from one experiment.

The findings that IL-7 signals appeared not to be important early during pMHC-stimulated LIP but were important when pMHC was limited (CiiTA deficient recipients), suggested the possibility that IL-7 becomes important when there is more competition for pMHC, such as in the post-LIP period. In splenocyte populations harvested at 5 weeks post-Marilyn thymocyte transfer, CTV labeling was virtually undetectable in all cells within the Rag^−/−^ female recipients (Figure 1B; Figure S1 in Supplementary Material). We reasoned that LIP was largely complete by this time given that we saw no further increase in absolute splenic CD4^+^ T cell numbers in mice harvested much later at day 76 post transfer (p.t.; data not shown). We, therefore, examined the phenotype and number of Marilyn cells late after cell transfer once T cell numbers had increased post-LIP and determined the effect of IL-7Rα blockade. A trend toward higher CD44 expression in PD-1^−/−^ versus WT cells was noted at 5 weeks post-transfer, which reached statistical significance in the anti-IL-7Rα-treated MHC-II sufficient females (Figure 3A). Importantly IL-7Rα blockade itself did not significantly decrease CD44 expression among T cells in female or male recipients, but MHC-II deficiency in females significantly reduced memory phenotype acquisition (Figure 3A). We also noted that Marilyn T cells chronically exposed to agonist antigen present in male recipients had greatly reduced CD44 expression compared to female recipients where only tonic pMHC signals were present. In contrast to the day 7 time point (Figure 2D), at day 35 both MHC-II sufficient female and male recipients receiving anti-IL-7Rα treatment had markedly reduced absolute splenic T cell numbers compared to their untreated counterparts (Figure 3B). As expected, MHC-II deficient female hosts had significantly fewer splenic T cells compared to their MHC-II sufficient counterparts (Figure 3B), and this was further reduced by anti-IL-7Rα treatment with very few cells detectable overall in anti-IL-7Rα-treated MHC-II deficient female hosts. Although most cells in non-anti-IL-7Rα-treated MHC-deficient females appeared to have undergone proliferation by day 35 a significant proportion remained undivided (Figure S1 in Supplementary Material). In contrast, the very few detectable cells within anti-IL-7Rα-treated MHC-II-deficient females were almost completely proliferated cells, consistent with the previously noted decrease in the undivided cell population upon IL-7Rα blockade (Figure S1 in Supplementary Material). We did not note any differential expression of IL-7Rα expression between WT or PD-1^−/−^ Marilyn T cells (Figure 3C) nor between cells in MHC-II sufficient or deficient female hosts although there was a trend toward lower IL-7Rα expression in the latter. Together, these data suggest that IL-7 signaling is dispensable for LIP in conditions of excess pMHC resources, but when the T cell compartment becomes more replete and competition for available pMHC resources increases or pMHC is otherwise limiting, IL-7 signals are critically important for survival of the T cell population.

### In a Competitive Environment Blockade of IL-7Rα Ameliorates Morbidity Caused by PD-1^−/−^ Thymocytes

**Figure 4:**
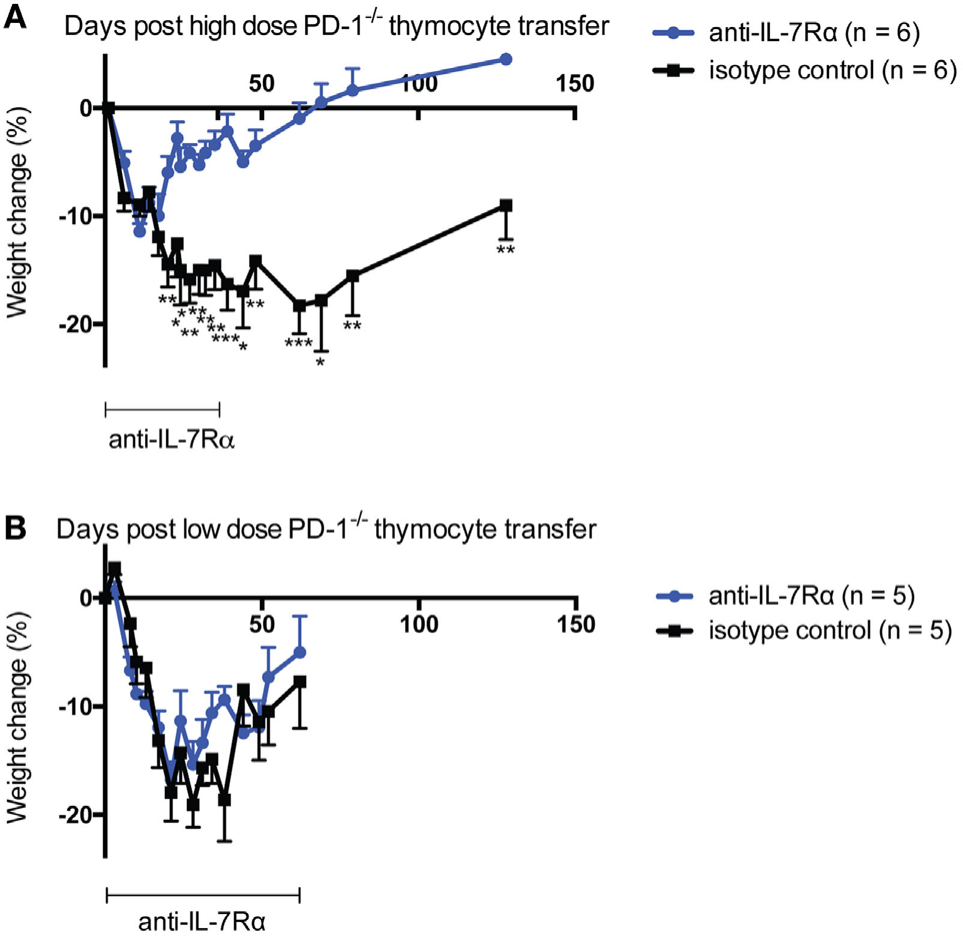
IL-7Rα blockade ameliorates high- but not low-dose PD-1^−/−^ thymocyte-mediated morbidity in lymphopenic recipients. Either high-dose [panel **(A)**, 30 × 10^6^ total cells] or low-dose [panel **(B)**, 10 × 10^6^ total cells] PD-1^−/−^ unfractionated thymocytes were transferred to NK-depleted Rag^−/−^ recipient animals with i.p. injection of 0.5 mg per mouse anti-IL-7Rα or isotype control twice weekly for the time periods indicated. Mice were monitored for disease symptoms and loss of weight. **(A)** Weight change in high-dose thymocyte recipients over the experimental period. **(B)** Weight change in low-dose thymocyte recipients over the experimental period. *p < 0.05, **p < 0.01, ***p < 0.001, Student’s *t*-test. The data depict the mean ± SEM of individual biological replicates from one experiment.

TCR signals can downregulate IL-7Rα expression (e.g., Figure 3C) and have been reported to block, in an affinity-dependent manner, the response to IL-7-mediated survival signals (49, 50). Because we noted that IL-7Rα blockade had a marked effect on the size of the T cell compartment 5 weeks post-mixed Marilyn T cell transfer (Figure 3B) but not after only 7 days in class II sufficient recipients (Figure 2D), we reasoned that IL-7 signals perhaps became more important to T cell survival as competition for available pMHC became more intense (i.e., as the T cell compartment became more replete, and fewer cells could sufficiently access pMHC and receive a TCR signal). In order to explore this concept, we examined whether constraints on IL-7 signaling in high versus low competition settings would influence the development of autoimmunity. For this we used our model of disease following transfer of polyclonal PD-1^−/−^ thymocytes to Rag^−/−^ animals, which results most commonly in weight loss, kyphosis, diarrhea, dermatitis, and ocular lesions. We transferred either a low amount (10 × 10^6^) or high amount (30 × 10^6^) of PD-1^−/−^ thymocytes to NK-depleted recipient animals. The recipients were treated biweekly with monoclonal anti-IL-7Rα or isotype control antibodies and monitored for weight changes and disease symptoms. Here, we noted a striking effect of anti-IL-7Rα treatment on loss of weight in the high-dose thymocyte recipient group, which was statistically significant from day 20 through to the termination of the experiment even though anti-IL-7Rα treatment was withdrawn at day 36 (Figure 4A). On the other hand, anti-IL-7Rα treatment of the low-dose thymocyte recipient group did not have any effect on the loss of weight (Figure 4B). Taken together, these data suggest that limitation of LIP potential by inhibiting the ability of T cells to respond to IL-7-mediated signals in situations of high but not low competition for finite pMHC can prevent the loss of weight associated with the pathology of LIP-driven autoimmunity.

## Discussion

The potential for LIP in a host depends on the resources available for T cells (tonic or cognate pMHC, homeostatic cytokines) relative to the level of competition for those resources (1). Importantly, LIP can drive inflammatory and autoimmune disease (7, 14, 17, 19, 22–30), and thus modulation of LIP can increase or decrease its potential to result in pathologic T cell activation. Several molecules with co-inhibitory function, such as BTLA, LAG-3, and TGFβRII have been shown to modulate T cell homeostasis and LIP (15–18), although to our knowledge none have demonstrated that such control of LIP *in vivo* is *via* modulating signals in response to pMHC, particularly tonic pMHC. Recently we described a severe systemic autoimmune disease mediated by newly generated PD-1^−/−^ T cells in lymphopenic animals which could be blocked by the addition of competitors or reducing lymph node stroma (19), and which was not due to a deficiency in the generation or function of PD-1^−/−^ Treg (33), suggesting that the co-inhibitory molecule PD-1 also plays an important role in the establishment of immune tolerance through control of LIP. This gave rise to our hypothesis that the disease in lymphopenic recipients of PD-1^−/−^ newly generated T cells is mediated by inappropriate activation of T cells in response to normally tonic self-pMHC in the context of LIP. However, the possibility existed that the T cell response in our disease model was directed toward tissue-restricted antigens that were not appropriately negatively selected against in the thymus in the context of PD-1 deficiency, or due to altered population dynamics because of changes in positive selection (51). Furthermore, it was unclear whether the relatively low-affinity interactions with tonic pMHC can promote upregulation of PD-1 on T cells for it to mediate its inhibitory function.

In female hosts, at least up to 7 days post transfer, PD-1^−/−^ Marilyn T cells showed greater proliferation (Figures 1B,C; Figure S1 in Supplementary Material) and came to significantly outnumber the Marilyn-WT cells by ~2-fold and this was not affected by IL-7Rα blockade but was completely dependent on host MHC-II expression (Figure 1D). This supports our hypothesis that PD-1 deficiency enhances the TCR signaling response to tonic pMHC during LIP.

When we examined the expression of the anti-apoptotic molecule Bcl2 at 5 days post-cell transfer (a time during which rapid LIP was occurring), the PD-1^−/−^ cells in female Rag^−/−^ hosts showed a trend toward expressing less of this molecule (Figure 2C). Although we did not examine other IL-7/TCR signaling-associated anti-apoptotic molecules, such as Bcl-Xl and Mcl-1 (52), this suggests that any potential increased survival in the PD-1^−/−^ population was not mediated by Bcl2. Importantly, examination of Bcl2 expression in T cells within all the recipient groups clearly indicated that Bcl2 expression was depressed in groups treated with anti-IL-7Rα and particularly in combination with pMHC deprivation in the CiiTA deficient hosts (Figure 2C). We also noted that at day 7, at which time cells were presumably still undergoing substantial LIP, IL-7Rα blockade did not reproducibly negatively impact the frequency of T cells in the blood in MHC-II sufficient hosts (Figure 2D). It is important to note that in contrast to studies that suggested that IL-7 signals were critical for the LIP of T cells (53, 54), anti-IL-7R treatment did not significantly inhibit proliferation in MHC-II sufficient hosts during the first week after thymocyte transfer when competition for pMHC would be expected to be low (Figure 1B; Figure S1 in Supplementary Material). The failure of T cells to undergo LIP in IL-7^−/−^ × Rag^−/−^ hosts (53, 54) is instead likely attributable to defects in lymph node structure and function as IL-7 is important for lymph node development (55). In contrast to our observation of no significant negative effect of anti-IL-7Rα treatment on the frequency of T cells in the blood of MHC-II sufficient recipients at day 7 post-transfer, IL-7Rα blockade led to significant decreases in absolute splenic T cell numbers by day 35 (Figure 3B). Together, these data suggest that by day 35, the recipients were more lymphoreplete and due to competition, tonic pMHC signals were no longer the most significant source for survival signals for the T cells and hence IL-7Rα blockade could now have a marked effect.

Despite its now clear role in controlling LIP in response to tonic pMHC-II signals, it was unclear whether PD-1 would be upregulated during LIP stably and sufficiently such that it would be detectable by flow cytometric staining. Indeed, at day 5 post-transfer, PD-1 expression was detectably increased by tonic pMHC signals in the WT T cell population but only among the highly proliferated cells (Figure 2A). The lack of obvious PD-1 expression on non- or intermediately proliferated cells (i.e., CTV mean fluorescence intensity ≥10^3^, Figure 2A) might suggest that regulation of LIP (and even perhaps normal primary T cell activation in a lymphoreplete host) by PD-1 may not require its high-level surface expression on T cells, or might occur primarily after many rounds of division in the highly proliferated cells. PD-1 upregulation may vary depending on the strength of the TCR:pMHC interaction experienced by a given T cell. Thus, the extent of PD-1 upregulation evoked upon LIP of Marilyn T cells in response to tonic pMHC may differ significantly from that of a polyclonal population (19) owing to a greater diversity of interaction affinities in the latter. Consistent with this notion, in the experiments described herein we noted that PD-1 expression levels were markedly lower among highly proliferated cells in female compared to male Rag^−/−^ recipients of Marilyn T cells (data not shown).

CD5 is a negative TCR signaling regulator commonly considered as a marker of TCR affinity set during thymic T cell selection processes as well as a reliable marker of TCR activation (45–47). CD5 expression has been correlated with the propensity for T cells from various TCR transgenic backgrounds to undergo LIP in a lymphopenic host (56–59). We found that highly proliferated cells stimulated by tonic pMHC in both the WT and PD-1^−/−^ Marilyn T cell populations contained subpopulations that had either up- or downregulated CD5 expression – with significantly higher overall CD5 expression in the PD-1^−/−^ group (Figure 2B). Potentially, CD5 is acting here as a readout of the extent of the TCR signal received by cells undergoing LIP. One could also speculate that the PD-1^−/−^ T cells are under pressure to maintain or increase CD5 expression due to a lack of co-inhibition from PD-1. However, pragmatically speaking these findings suggest that CD5 expression can change considerably in a monoclonal T cell population during LIP, which raises important questions about the validity of its use as a marker of T cell affinity/avidity for thymic pMHC as commonly seen in the literature, particularly if the cells under analysis have been exposed to a lymphopenic environment and have undergone LIP.

Compared to its effects on LIP in response to tonic pMHC signals, the effect of PD-1 deficiency on LIP of Marilyn T cells was even more pronounced in response to cognate pMHC in male Rag^−/−^ hosts (Figure 1D) and it was also IL-7 independent. In CiiTA^−/−^ hosts, APC within the eye and brain can use a CiiTA-independent pathway to express MHC-II in response to IFN-γ and TNF-α (44), and it is possible that low numbers of MHC-II sufficient thymic APC were co-transferred with the Marilyn thymocytes. While robust proliferation occurred in male CiiTA^−/−^ × Rag^−/−^ hosts, interestingly no appreciable increase in the PD-1^−/−^:WT ratio was seen by day 7 post transfer. While further studies are needed to fully understand this difference, two possibilities seem likely. Either the residual MHC class II expression in the CiiTA^−/−^ recipients is limited to specific APC types that present antigen but do not engage PD-1 or alternatively it is simply the quantity of antigen exposure that determines whether PD-1 controls the response. We have previously proposed that chronic antigen signaling, as would occur with widely distributed pMHC ligands, leads to upregulation of co-inhibitory signals (60). From this viewpoint, the intermittent (not chronic) exposure to sparse pMHC in female or male CiiTA^−/−^ × Rag^−/−^ recipients would not lead to co-inhibitory signals such as those through PD-1.

Although we did not note any difference in IL-7Rα expression between WT and PD-1^−/−^ T cells (Figure 3C), we did note that compared to female hosts, the IL-7Rα-expressing T cell populations in male recipients were considerably less numerous which is consistent with these cells constantly receiving strong TCR signals from high-affinity cognate pMHC interactions and downregulating IL-7Rα. Chronic high-level stimulation may also underlie the decreased CD44 expression noted in male MHC-II sufficient hosts (Figure 3A), a phenomenon previously observed in chronic viral infection (61). Indeed, the much larger size of the T cell compartment within male versus female hosts (Figure 3B) stems from the presence of pMHC that can provide a higher affinity signal (i.e., cognate pMHC) and could be viewed as a richer source of resources. Thus a HY containing host was able to support a much larger population of T cells.

Consistent with our interpretation that increased competition for pMHC signals in more lymphoreplete conditions would create greater dependence on IL-7Rα signals, we found that IL-7Rα blockade had a robust effect on preventing and reversing weight loss only when high numbers of PD-1^−/−^ thymocytes were transferred (Figure 4A). IL-7 has previously been associated with the promotion of IL-3 and GM-CSF expression in human T cells (62). Very recently, it was reported that IL-7 could promote the development of a unique subset of GM-CSF and IL-3-producing T cells (“Th-GM”) in mice and this was associated with encephalitogenicity in the experimental autoimmune encephalomyelitis model (63). Based on these reports, we considered that the effects of IL-7Rα blockade might be attributable to inhibition of GM-CSF expression and, therefore, we explored this *in vivo via* administration of anti-GM-CSF antibody to lymphopenic recipients of PD-1^−/−^ HSC. We found a small, statistically insignificant effect of anti-GM-CSF blockade in this experiment on disease incidence with no significant effect on loss of weight (Figure S2 in Supplementary Material). These data suggest that the effect of blockade of IL-7Rα is unlikely to be solely mediated through effects on GM-CSF. Our findings support the concept that in situations of high competition for pMHC, limiting LIP potential by blocking the response to homeostatic cytokines like IL-7 can help to ameliorate the wasting aspect of systemic autoimmunity.

Our finding that the co-inhibitory molecule PD-1 controls LIP by modulating the response to tonic self-pMHC signals *in vivo* lends support to our hypothesis that the disease in lymphopenic recipients of PD-1^−/−^ HSC or newly generated T cells results from exaggerated responses to normally tonic self-pMHC signals. These data are consistent with the concept that establishment of peripheral tolerance involves a tuning process (64), a tuning that is regulated at least in part by PD-1. Furthermore, the effects of PD-1 deficiency on control of LIP were independent of IL-7Rα-mediated signaling, and IL-7-mediated signals were largely irrelevant to the size of the overall T cell compartment while LIP potential was still high and rapid LIP in response to abundant pMHC was occurring within the hosts. These data suggest that in LIP-associated inflammatory disorders, such as immune reconstitution inflammatory syndrome in HIV patients (25, 65), or graft-versus-host disease, therapies aimed at reducing TCR signaling during early phases of reconstitution may be more effective and should take priority over approaches that aim to limit homeostatic cytokine-mediated signals to T cells. Furthermore, the ability of PD-1 to control tonic signals in response to self-pMHC may partially underlie the effectiveness of PD-1 blockade in tumor immunotherapy—for example, by enhancing survival and activation of T cells in the context of limiting neoantigen-derived pMHC in the tumor microenvironment or by promoting tumor clearance through the enhancement of collateral damage (43).

## Ethics Statement

This study was carried out in accordance with the recommendations of the Canadian Council on Animal Care. The protocol was approved by the University of Alberta Health Sciences Animal Care and Use Committee.

## Author Contributions

KE designed, performed research and data analysis, and wrote and critically edited the manuscript. JL performed research and data analysis and critically edited the manuscript. LB provided reagents and critically edited the manuscript. CA designed research, performed data analysis, and critically edited the manuscript.

## Conflict of Interest Statement

The authors declare that the research was conducted in the absence of any commercial or financial relationships that could be construed as a potential conflict of interest.
