# Supplementary figures and images for: PD-1 Controls Tonic Signaling and Lymphopenia-Induced Proliferation of T Lymphocytes
Source: Front Immunol. 2017 Oct 12;8:1289. doi: 10.3389/fimmu.2017.01289 (PMC5643416; doi:10.3389/fimmu.2017.01289)

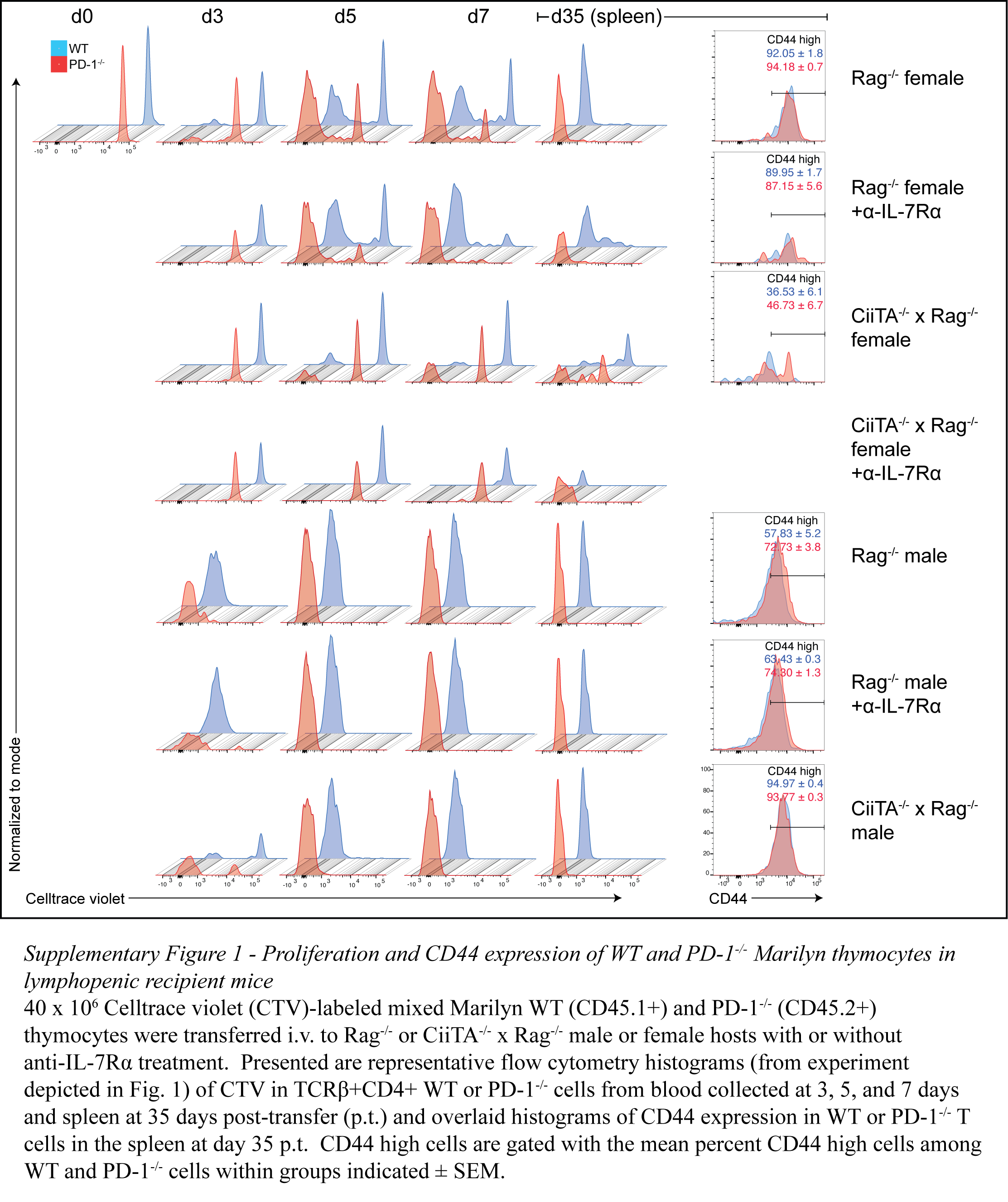

Supplement: Supplementary file 1 [file Image_1.TIF]

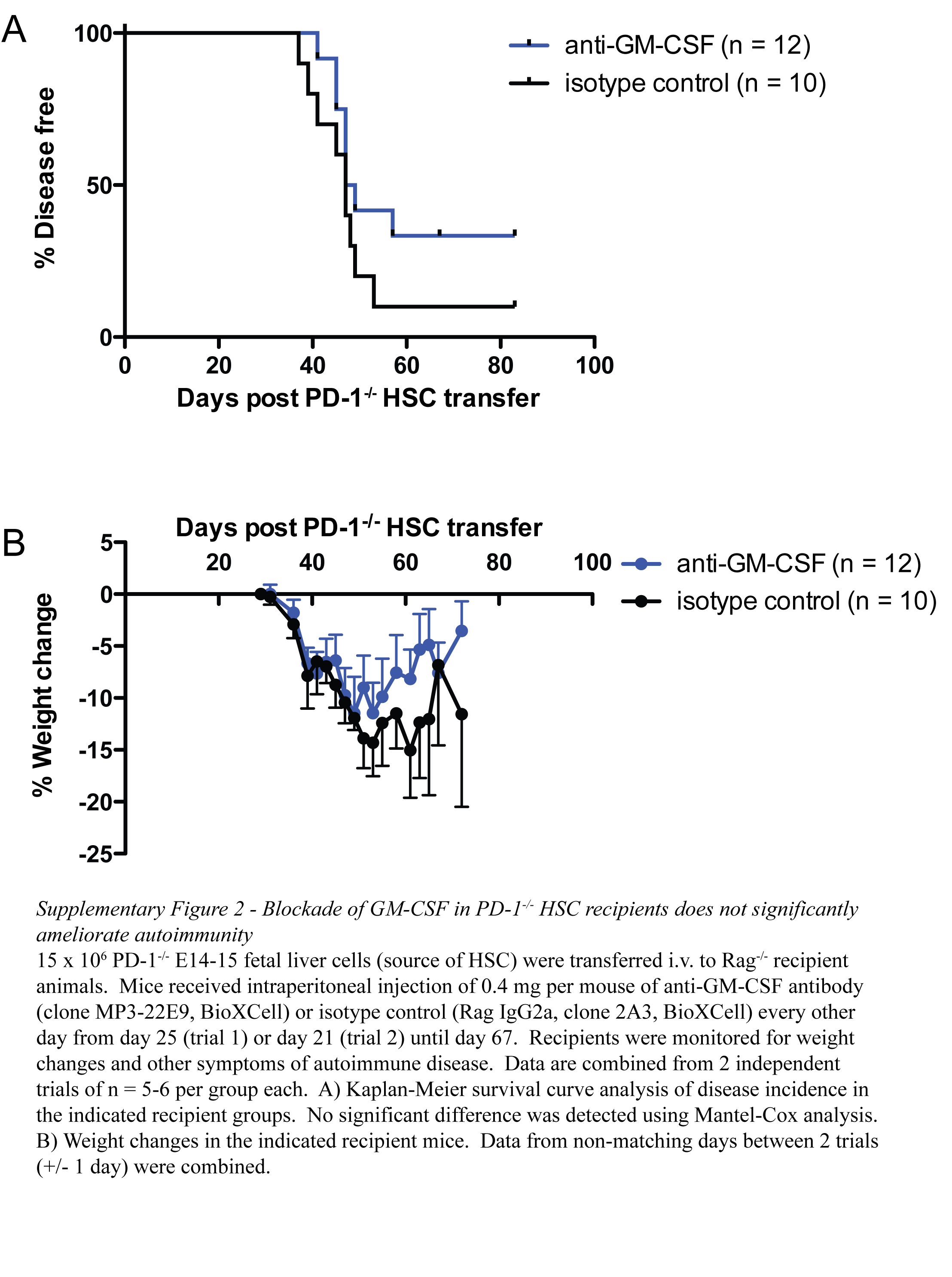

Supplement: Supplementary file 2 [file Image_2.TIF]
